# Interaction Design Patterns for Augmented Reality Fitting Rooms

**DOI:** 10.3390/s22030982

**Published:** 2022-01-27

**Authors:** Pietro Battistoni, Marianna Di Gregorio, Marco Romano, Monica Sebillo, Giuliana Vitiello, Alessandro Brancaccio

**Affiliations:** 1 Department of Computer Science, University of Salerno, 84084 Fisciano, SA, Italy; pbattistoni@unisa.it (P.B.); madigregorio@unisa.it (M.D.G.); msebillo@unisa.it (M.S.); gvitiello@unisa.it (G.V.); 2 Youbiquo s.r.l., 84013 Cava de’ Tirreni, SA, Italy; alessandro.brancaccio@youbiquo.eu

**Keywords:** augmented reality, meta-user interfaces, user experience, usability

## Abstract

In this work, we explore the role of augmented reality as a *meta-user interface*, with particular reference to its applications for interactive fitting room systems and the impact on the related shopping experience. Starting from literature and existing systems, we synthesized a set of nine interaction design patterns to develop AR fitting rooms and to support the shopping experience. The patterns were evaluated through a focus group with possible stakeholders with the aim of evaluating and envisioning the effects on the shopping experience. The focus group analysis shows as a result that the shopping experience related to an AR fitting room based on the proposed patterns is influenced by three main factors, namely: the perception of the utility, the ability to generate interest and curiosity, and the perceived comfort of the interaction and environment in which the system is installed. As a further result, the study shows that the patterns can successfully support these factors, but some elements that emerged from the focus group should be more investigated and taken into consideration by the designers.

## 1. Introduction

Today, given the spread of IoT services, the presence of smart environments is increasingly common in all areas of daily life [1]. For such reason, people are already used to interacting with the environment through dedicated and easily accessible services. For example, personal assistants such as Google Assistant (assistant.google.com, accessed on 10 December 2021) or Alexa (developer.amazon.com/alexa, accessed on 10 December 2021) are increasingly becoming part of daily environments and allow people to control electronic connected devices in the environment or receive information from sensors.

Services and related interfaces that allow people to interact with the surrounding environment are commonly called *meta-user interfaces* [2].

In the context of *meta-user interfaces*, augmented reality (AR) can play an important role because of its nature in dealing with both digital and physical worlds together. AR allows us to digitally increase the surrounding environment and therefore can be designed to allow users to intuitively access digital services distributed in the environment.

AR has been receiving great attention in the industrial field in recent years. Its global market, indeed, has been estimated at around 15 billion USD and is expected to grow at a CAGR of 31.5% by 2026 (www.marketsandmarkets.com, accessed on 10 December 2021). Sales business sectors are increasingly interested in leveraging this technology in various forms to broaden their market, using it as both a marketing tool and to offer new engaging utilities to their consumers [3].

A sector at the forefront of the usage of AR technology is the fashion retail industry [3,4,5]. Customers have, indeed, been accustomed for years to using AR mobile applications to try on makeup or to take augmented photos with virtual clothes. When devices enabling people to try on virtual garments are in malls or stores, they are commonly called magic mirrors.

A magic mirror is a public display that can be identified by users as a normal mirror able to augment the space surrounding the user or the user himself. It was used in advertisement campaigns of famous brands [6], such as: Pepsi, in which the mirror transforms people’s faces into evil clowns or werewolves; National Geographic, where people walking in a mall can interact with wildlife appearing around them; and Timberland, which allows people to try clothing and shoes using digital screens in shop windows.

In literature, the terms magic mirror and AR fitting or dressing room are often overlaid. However, when the magic mirror is part of a system specifically aimed at providing customers with an effective and alternative tool to try on garments, we refer to that system as an AR fitting room.

Nevertheless, this technology, despite its use becoming more and more common, still needs efforts to be effective. Indeed, in [7] the author explains that a real and complete interaction between users and digital AR contents may be technologically difficult to develop and many marketers give consumers just a fake perception that they are wearing AR garments while replacing customers’ body with avatars arranged in advance.

Additionally, as stated in [8,9], it is not enough to develop a functioning magic mirror; it is important that designers provide AR systems that give added value to the experience and encourage customers to use them. This is particularly true when the users are consumers of a store and the use of the magic mirror depends solely on their interest.

Most studies focus mainly on technical aspects of implementation, although the main motivation is the improvement of the shopping experience as in (e.g., [10,11,12]). For such reason, starting from usability principles and the analysis of existing interactive fitting room systems present in the scientific literature or already on the market, we propose a set of interaction design patterns to develop efficient AR fitting rooms able to enhance the shopping experience. The patterns were used to prepare case studies, which were submitted to a group of people participating in a focus group designed to evaluate the potential shopping experience and the factors that influence it.

From this focus group activity, it emerged that the shopping experience of the AR fitting room is linked to the following factors: perception of the utility of the system, the ability to generate curiosity and interest, and the interaction and comfort in the environment. The study shows that the patterns can successfully support these factors, but some elements that emerged from the focus group should be more investigated and taken into consideration by the designers.

The rest of the paper is structured as follows: Section 2 describes the analyzed interactive fitting room and related systems present in the literature or in use on the market and discusses some good practices when designing AR systems. Section 3 shows typical architectures that allow magic mirrors to be developed with the technology available today. Section 4 presents the interaction design patterns, and Section 5 presents and analyzes the focus group to assess their impact on the shopping experience. Finally, in the last section, some conclusions and final remarks are given.

## 2. Related Work

In this section, we present the existing interactive fitting rooms and related best practices for designing AR systems that were considered in structuring the proposed patterns.

KinectShop by Razorfish is based on a Kinect (developer.microsoft.com/it-it/windows/kinect, accessed on 10 December 2021) camera and a TV and allows users to try virtual items via AR. The interface is completely gestural; on the side of the screen, a vertical stripe with the products is present. The user can slide it up or back and select a product by grabbing it through a “close fist” gesture [13].

Bodymetrics is a virtual dressing room that allows the user to see how she wears a dress in a shop or at home [13]. A virtual mannequin, based on the user’s appearance, continuously follows the person’s movements. The interaction is gestural; the flow is as follows: (1) The user brings his hand forward to activate the interaction stage. As a reaction, a shadow hand appears, which will follow the user’s real hand. (2) Keeping the hand still on an interactive object activates it. (3) By touching the end of the strip containing the catalog, the strip scrolls up or down. (4) A series of buttons allows the user to choose size or color. (5) Lowering the hand ends the interactive stage.

WSS For Kiosks is a Microsoft Kinect-based AR fitting room and large public display [14]. It allows users to measure a set of clothes. This is a fully gestural interface. The interaction flow is as follows: (1) The screen shows an empty template that the user has to enter to take the measurement. When the measurement starts, the shape begins to fill with color. (2) Once the measurement is finished, a button interface appears around the user. Users have to stretch out the arms and touch the buttons with the hands for a couple of seconds to confirm the selection. (3) The top button allows users to select the type of garment (hat, pants, t-shirt, etc.). It must be touched several times, as a “tap”, to change the type of garment. (4) The buttons at the bottom allow users to scroll through the catalog, appearing in a stripe on the top, and they see the garment appear directly on them.

Uniqlo Color Change Mirror allows a user who physically wears a garment in the store to see the same garment in different colors [15]. The interaction is very simple: after a short time, the real garment changes color in AR. The advantage is to give the user the real feeling of wearing the garment. A companion app is used to allow consumers to control the color change and drive the experience.

Kang [16] presets a study about using magic mirrors at home to try garments before buying them. The study performed with potential online clients shows that using a magic mirror in an online shop increases the intention to buy and engage the clients. The magic mirror used in the study uses a webcam to scan and track the users’ bodies and their movements via motion capture and allows them to put on various virtual garments as they would in a fitting room. The computer screen becomes an augmented reality mirror where the users can see how the clothing might look in real-time and browse various garments without a keyboard and mouse. Users can also adjust the size and color of the clothing item simply by using hand gestures.

The interface is based on a dual interaction: gestural- and mouse-based. The left part of the interface is based on body and gesture recognition. The system requires the user to stand in front of the camera so that they can be measured and identified. It offers a visual interface based on buttons positioned around the fitting area and therefore the user. The user will then be guided in the gestures by touching the buttons on the screen with the index finger. Using the buttons, it is possible to change the style and color of the product. The right part of the screen is dedicated to the catalog, and users can interact using a mouse pointer.

The authors of [16,17] propose a system dedicating a specific space to the catalog and the fitting. Here, the authors present an interface based on two Kinect cameras and two screens. One screen is dedicated to the catalog presented in the form of a virtual show room and the second screen to the AR fitting room. Then, users select the garments in the first screen using gestures recognized by the first Kinect and see how they fit in the second screen where the second Kinect recognizes the body.

In the virtual show room, users interact by hand gestures; the left hand is used to select the garments and the right to confirm the selection. Two hand shadows follow users’ hands, and they are in two different colors to make it easier to users to understand the right and left hand and associate the different interactions’ meanings (selection and confirmation).

In these two last examples, the researchers clearly separated the two phases of the fitting room interaction. One kind of interface is dedicated to the catalog by dividing the screen or using two screens, and one kind is dedicated to the fitting.

Other products not specifically available on the market today may be of interest to those who design AR fitting rooms.

The Smart Fitting Room by Adidas [18] is not an AR system but allows clients to get more information about the product they are trying in a fitting room and lets them ask for help. The interaction starts by putting the product close to the fitting room mirror. The system recognizes the product using a touchless technology, such as Radio Frequency Identification (RFID). Then the mirror displays an interface with product details and the possibility to ask an assistant to bring in the fitting room another size or color of that product.

Nike’s AR shop window [19] is not a mirror for trying on clothes but provides interesting details regarding the interaction with an AR showcase. The shop window reacts to people’s walking, playing animations that follow people’s movements. A sticker on the sidewalk invites people to stop in front of a specific point in front of the shop window to start the interaction. The system allows a body interaction, for example, the user can jump, or gestural. In this case, the user is asked to touch the shop window, this is a mid-air gesture for the system but gives the user the realistic sensation of touching a physical button.

### Good Practices

Literature highlights, on the one hand, some common interaction problems of magic mirrors on the market and, on the other hand, some good practices to take into consideration:

**Pay attention to the “Gorilla arm syndrome”.** The gorilla arm syndrome originally arose with the advent of touch and mid-air gestural interface, which force users to extend their arms without support. This can cause arm fatigue and a feeling of heaviness in the arms [20]. This does not mean that mid-air gestures should always be avoided [21]. Gestures performed vertically (with the arm extended above the heart) are certainly more tiring than gestures performed horizontally (at or below the level of the heart). Therefore, it is necessary to avoid repeating continually vertical gestures unless you provide support for the arm. This is to support the effectiveness of the interaction and the user experience.

**Avoid overcrowding the user interfaces.** One of the main problems that can be encountered when designing augmented reality systems is overcrowding the user interface with graphical elements [22,23,24]. The creative temptation to design highly engaging digital experiences is common; however, it must be remembered that in augmented reality experiences, users use digital content to complement their reality and not to obscure it. Users overloaded with information and being in an uncontrolled space could even perform dangerous actions for themselves and for the people around them.

**Interacting in steps.** In general, to make the interaction usable, it is necessary to provide users with separate interaction steps for each task. It allows users to focus on one task at a time and not be distracted by other factors. This approach is widely used in the design of mobile applications where a specific view is dedicated to each main task [25]. However, this type of approach can be adopted whenever an interactive system requires users to effort and focus on tasks.

As seen in some systems of AR fitting rooms [16,17], designers prefer to clearly divide the users’ tasks into different steps.

This brings multiple benefits to the user experience of this type of product. Users can dedicate attention and efforts to complicated tasks, such as garment selection and body measurements, while they can enjoy the main task, which is seeing the AR result and playing in front of the mirror.

**Mixing digital and physical interaction.** We, as human beings, are used to perceiving and interacting with the surrounding environment using different senses. When we talk to someone, we use our voice, we hear it through our hearing, we express ourselves simultaneously with our eyes and facial expressions, and we gesticulate and adopt a certain position [26,27].

For this reason, interactive systems that involve multiple senses are perceived by users as more engaging, natural, and therefore familiar [28,29]. The interfaces supporting such systems are called “multimodal” [30]. The authors in [31] strongly recommend designing AR interfaces for the senses of users, not for the devices the system will run on. This is valuable advice because it invites designers to focus first on experience and then on technology, ensuring that every design choice is made first in the interest of the users.

This point of view guides AR designers not to focus only on the digital experience but to exploit more the real world as well. In an AR system, touching physical objects, such as stickers or products, making the real environment part of the experience, receiving tangible feedback, for example through vibrations, sounds, or voice, contribute a lot to the development of a good user experience.

**Blending AR-enabling objects into the environment.** In [32], the authors explain that the enabling objects of augmented reality must be able to blend into the surrounding environment as much as possible so that they appear to be part of the user’s ecosystem.

In this context, they introduce the augmenting smart objects (ASOs) concept as a way to support engaging experiences. An ASO is a nonintrusive and interactive device allowing users to access and interact with AR content in different contexts with a view to creating personal links between visitors and the environment. The basic idea is that these objects, appearing as sewn into the environment, do not have to completely capture the attention of users: they must facilitate users’ tasks and allow them to focus on the real environment.

## 3. Hardware and Architectures

It is possible to build a relatively simple hardware system similarly to that demonstrated by [33], but in order to give users a better experience it is necessary to build more complex architectures, allowing for clarity of image and space for interaction. Most importantly, all architectures need to collect data about the user to precisely calculate his/her body proportions. Therefore, the use of depth cameras systems capable not only of retrieving an RGB image of the user but also a depth image with precise distances is required for building such systems. The depth image should cover most of the user’s body to be effective; therefore, in the following architectures, a system comprised of two depth cameras is always implied.

Another important point is that while state-of-the-art measuring systems such as Naked (nakedlabs.com, accessed on 10 December 2021) are already available, they provide an entirely static experience for the user. To make the interaction more immersive and responsive to the user, it is necessary to have a real-time virtual clothing experience.

**Figure 1:**
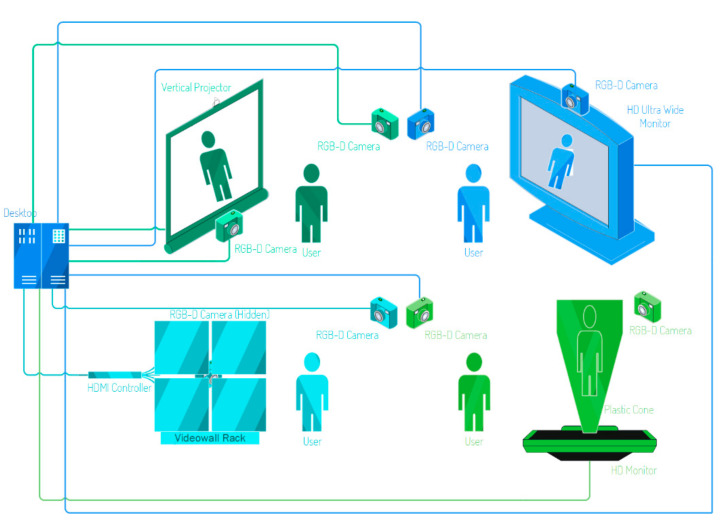
Different magic mirror setups side-by-side.

Finally, providing a real-time fitting experience requires powerful hardware—either available locally in the fitting room or on cloud resources—to handle all the necessary computations. With these premises, a variety of hardware configurations are possible, as shown in Figure 1 below.

### 3.1. Computational Hardware and Cameras

For the body recognition and body tracking calculations, it is strongly suggested to use desktop hardware as described in Table 1. While many other computational units are available, such as Nvidia’s AGX Xavier (www.nvidia.com/it-it/autonomous-machines/embedded-systems/jetson-agx-xavier, accessed on 10 December 2021) or Intel’s Neural Compute Stick (www.intel.com/content/www/us/en/developer/tools/neural-compute-stick/overview.html, accessed on 10 December 2021), they are often specialized for intensive machine learning contexts and come with significant drawbacks. This is the case of the Neural Compute Stick, which does not rely on any Operating System (OS), or of the AGX Xavier, which is bound to Linux OS for Advanced RISC Machines (ARM), making it impossible to use more popular application development IDEs such as Unity or Unreal Engine.

Desktop components offer a wide variety of specifications that can be used to better suit the need of the software being run; therefore, components remain easily accessible, even during chip shortages.

Specialized depth cameras can be used to further enhance measurements. In particular, cameras such as the OAK-D (https://store.opencv.ai/products/oak-d, accessed on 10 December 2021) can provide edge computing synergizing well with the desktop hardware, while stereo depth cameras such as Realsense D435i (www.intelrealsense.com/depth-camera-d435i, accessed on 10 December 2021) can provide high-quality depth data in a compact way.

### 3.2. Systems

It is easy enough to build an AR fitting room system as described by [34]. Nonetheless, such systems require a costly calibration needed to correctly set up the camera in relation to the user.

#### 3.2.1. Videowall System

If physical constraints make it possible, a videowall system can be a flexible solution for many cases, either in a private fitting room or in the shop display window.

As shown in Figure 1, the modular borderless screen panels arrangeable in diverse matrixes allow for fine tuning of screen space relative to available space, while also offering space for installation of the hardware components needed to run the measurements algorithms. The required depth cameras can be installed beside the user and directly in front of the user by hiding the camera in the videowall seams.

#### 3.2.2. Hologram System

A novel approach to the user experience can be derived from the paper by [35]. In the paper, a transformation of the image output by the screen can be projected on an arbitrary surface. While the experiment scale does not cover full-human-size, similar systems have been used during concerts in very effective ways, as demonstrated by BASE Hologram (https://basehologram.com/experience#312-ourDifference, accessed on 10 December 2021).

**Figure 2:**
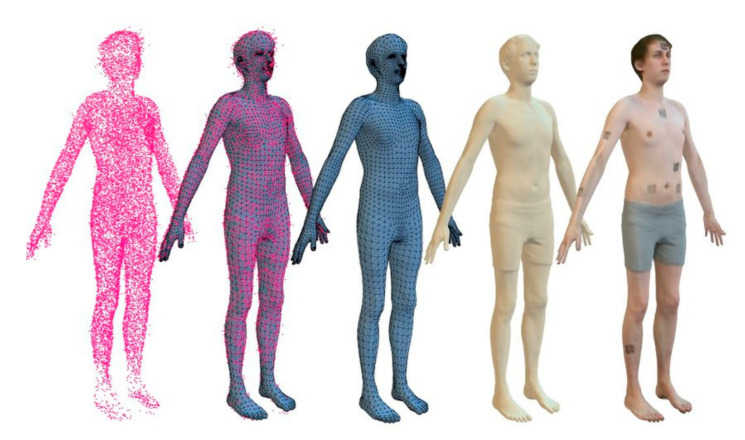
Steps for mesh reconstruction.

In this particular setup, illustrated in Figure 1, the depth cameras need to be oriented toward the user, thus introducing a necessary calibration step. The novelty of the approach can also influence how the tracking is done. Trading real-time tracking for a one-time scan tracking approach can allow for the reconstruction of the user’s 3D model, as shown in Figure 2. This can be achieved by detecting the cloud of points of the user, reconstructing the user’s mesh using Delaunay’s triangulation algorithm or derived approaches, as described by [36].

#### 3.2.3. Projector System

A system that shows the user image through a projector can be very space efficient and of impact to the user. Practical commercial uses have already proved the maturity of this technology, as done by Lymb.io (Lymb.io, accessed on 10 December 2021).

Ultrashort projectors can apply very high 4K resolutions while being mounted directly under or above the surface where the image is projected. Mobility and easiness of installation are the doubtless advantages of such a solution. The downsides are found mainly in the ambient light and general cost of the solution. Such projectors cost thousands of euros, while a strong ambient light can have a negative impact on the quality of the projected image. Nonetheless, such a system can be useful in specific cases.

## 4. Interaction Design Patterns to Build AR Fitting Rooms

Starting from the analysis of existing solutions of AR fitting rooms, magic mirrors, and other similar smart products used in retail and scientific literature, we identified and structured nine interaction design patterns corresponding to the common AR fitting room activities:

1. Body measurement;
2. Intuitive and comfortable interaction;
3. Selecting the garment’s characteristics;
4. Selecting the garment’s characteristics automatically;
5. Selecting a garment in the virtual catalog of a store;
6. Selecting a garment in the virtual catalog of the store using a second device;
7. Physically selecting a garment in the store using a smart wardrobe;
8. Physically selecting a garment in the store using a mobile device;
9. Magic shop window.

Patterns 3 and 4 address a similar problem and are alternative to each other; the same is true for patterns from 5 to 8. Each pattern is structured according to [37] using the following scheme: Title, Problem, Context (To be used when), Solution, and Example. Table 2 gives a brief explanation of each field in the structure. The solutions were built on the basis of usability principles presented in [38,39] and good practices in the previous section.

These patterns, after being structured, were discussed with a team of developers and engineers in AR systems development to verify their technical feasibility.

In particular, we performed the following steps:

**Step 1:***Identification of the main activities in an AR fitting room system*

In this step, researchers from the HCI laboratory of the University of Salerno and engineers of Youbiquo Company, which are experts in AR technologies, identified the main activities of the interaction between customers and an AR fitting room based on the state of the art of technology, experience, and literature.

The identified activities are: body measurement, selection of the garment to wear, and playing in front of the mirror. Furthermore, three possible locations to use the fitting room were identified: a traditional fitting room, an open space dedicated in the shop, and a shop window.

**Step 2:** *Identification of patterns and usability principles*

Through a formal study of the literature and existing systems, researchers identified recurrent interaction patterns relating to the phases in the previous step. Furthermore, usability and UX principles related to them were analyzed to define and structure nine interaction design patterns.

**Step 3:** *Reviewing the interaction design patterns*

The interaction design patterns were reviewed with the Youbiquo engineers to study their technical and practical feasibility. This made it possible to improve the patterns making their development more feasible.

**Step 4:** *Scoring the strength of evidence*

The researchers evaluated the proposed patterns on the basis of the *strength of evidence*. The *strength of evidence* is a scale indicating which patterns designers can place the greatest confidence in or should pay more attention to during usability testing [40]. It is rated with a score ranging from 1 to 5, where 1 is the minimum score, and 5 is the highest. The scores were assigned by researchers on the basis of the use cases found in literature or on the market and the applied usability principles or performed tests on them.

Table 3, Table 4, Table 5, Table 6, Table 7, Table 8, Table 9, Table 10 and Table 11 describe each pattern in detail.

## 5. Studying the Effects on the Shopping Experience

To assess the impact of the patterns on the shopping experience, we prepared a focus group with people representing potential stakeholders. We adopted a qualitative research approach because, as shown in [41,42], a focus group is suitable to explore potential experience, specific elements affecting it, and to generate new ideas and perspectives.

In particular, we were interested in exploring the perception of usability and utility in a set of interaction scenarios adopting the described patterns and their impact on the shopping experience. Such an approach was selected among others to also allow stakeholders to envision new ways to use AR fitting rooms to improve their shopping experience.

### 5.1. Procedure

Due to local and academic restrictions, the activity took place remotely using Google Meet (https://meet.google.com, accessed on 10 December 2021). This also provided the opportunity to involve experts and stakeholders situated in different locations.

The whole session lasted about two hours, including a ten-minute break and an explanatory introduction.

The activity was divided into four stages: (1) profile questionnaire and personal presentation, (2) introduction to some graphical and video examples of the applications of the patterns, (3) discussion, and (4) greetings and thanks.

In the first stage, the participants were required to fill out a questionnaire about their profiles. They were asked about their profession, age, shopping habits, and their technological knowledge.

Furthermore, one researcher acted as a facilitator, and another was in charge of observing and taking notes. The whole session was recorded to allow researchers to transcribe the focus group and review some behaviors.

### 5.2. Participants

The focus group activity involved a group of heterogeneous stakeholders, precisely, 10 participants (M = 4, F = 6) with a mean age of 36.6 (SD = 9.4). They were a corporate management expert, a salespersons manager of a well-known European clothing chain, a sociologist expert in the field of sales, a marketing practitioner, a computer engineer, an innovation manager, and, finally, four participants without technical specializations to have points of view without biases.

All the participants described themselves as technologically proactive. Just one of them bought clothes exclusively in physical stores during the last year, and, in general, all of them declared that they use technology as support when shopping, for example, to involve a friend by sending photos and asking for opinions, compare prices from other online catalogs or to check the availability of a product in a store.

The heterogeneity of profiles allows us to have different points of view covering both the technical and nontechnical perspectives as well as business and customer points of view.

### 5.3. Questions and Stimuli

During the focus group activity, images, videos, stories, and drawings made at the time were used as stimulus material for discussion and to introduce augmented reality technologies, the AR fitting room, and the various cases where the patterns were used.

The facilitator shared his screen with the participants and was supported by an additional stylus-enabled screen.

The facilitator presented, one by one, a case study where a design pattern was applied. The case study was presented through images and videos. After each presentation, the facilitator asked questions, allowing all participants to answer.

Focus group questions were open-ended to allow flexibility and encourage discussion [42]. Examples of the included questions were:

- *What is the role of technology in your shopping activities?*
- *Given an AR fitting room, as in the example, do you think you would be able to use it?*
- *Given an AR fitting room in the example, can you envisage how and when to use it during your shopping?*
- *Given an AR fitting room in the example, what do you think about its effects on your shopping experience?*
- *What would be helpful to include in an AR fitting room to improve your shopping activity?*

Further prompts were used to clarify and extend concepts when needed.

### 5.4. Comments and Reactions from the Participants

In general, the group expressed a positive opinion on the usability of all the cases presented. The opinion that a magic mirror is easy for a child or an elderly person was commonplace for everyone. Using stickers to guide while interacting with the magic mirror, combined with digital instructions, were compelling elements for the positive perception of usability.

However, regarding the measurement case, the participants expressed some concerns related to the shopping experience, especially when the magic mirror is not placed in a covered fitting room but instead in the shop window or common space in the shop.

One participant said, “*where should I put my coat or shopping bags to perform the measurement?*” Another added, “*this system easily draws the attention of shoppers and passers-by; they would observe me while measuring creating a privacy problem*”. One more participant said, “*While they are intuitive, these initial steps may discourage customers from using the AR system*”.

The group worked on the raised doubts, proposing some shared solutions. Regarding the coat or shopping bags, a magic mirror should always be prepared with easy-to-reach shelves and a coat hanger. In relation to the privacy issue, they proposed that the measurement can be done optionally via an app at home so that it can be used in affiliated stores. Regarding the initial discouragement, a shop assistant present in the surroundings of the magic mirror can give confidence to those who use the system for the first time.

Finally, the measurement activity was generally considered useful by all, as it helps customers find the right size immediately by speeding up the shopping activities.

Although the virtual catalog was considered usable, the participants proposed some alternatives that would make the interaction more natural. One said, “*I would prefer a variety of more natural gestures, such as dragging a digital hat to my head or a shirt across my chest*”, and another said, “*A greater variety of gestures would be helpful, not just making the fist but for example indicating the garment to fit. It would help people who don’t know the exact interaction predict it based on other experiences*”.

Finally, one said, “*I would like to have sensory feedback when I select a dress, such as haptic to make me feel the AR experience as more natural*”.

The virtual catalog was considered a useful tool able of speeding up shopping activities and supporting customers when it is necessary to change color or size or when they look for another model to wear without having to ask for help from others or leave the dressing room.

Regarding the use of a second screen based on touch interaction, users did not find it particularly useful compared to the virtual catalog. The common perception was that the system could be perceived as more complex by some customers: “*a second device can discourage its use by making the whole system perceived as complex*”.

However, an additional touch screen could be a viable alternative for customers unfamiliar with mid-air gestures. Then, the group suggested a tablet device to be provided on request.

The smart wardrobe was the only case in which the group dynamics were divergent. The group split in half on its utility and benefits on the shopping experience.

The criticisms were: “*I would spend more time looking for the product to wear*”; “*Given the health situation, some people may prefer not to touch the products*”; “*It’s easy, but I find bringing the clothes to wear uncomfortable*”; and *“It can incentivize people to take lots of clothes for fun and then abandon them, causing difficulties for the store*”.

On the other hand, the rest of the group found benefits for the shopping experience: “*The advantage is that you realize what you are buying. You can see and touch the real garment and understand if you really like it*”; “*You wear it virtually without wasting time undressing*”; “*It increases the interactions with the product, and it can make the more pleasant experience*”; and “*It can be slower to find the product, but I would use it if I’m not in a hurry*”.

The distinct perceptions lie in the fact that the system is aimed at enhancing the shopping experience by involving the full human senses, such as touch and sight, at the expense of practicality. Then, these considerations suggest the use of this case to enhance the experience of particular customers and not to speed up shopping.

Physically selecting a garment in the store using a mobile device appeared to the participants as the right compromise between practicality and experience. The comments were: “*It’s practical, you can touch or not as you like and then you go to the mirror*”; “*There is no risk of leaving clothes around the store to play with the mirror*”; “*It is engaging thanks to the possibility of touching real products*”; “*I prefer a mixed experience between digital and physical. Having the ability to touch involves people more*”; “*You don’t lose the reality of the store and therefore the sense of going there*”; and “*Quicker and more comfortable than bring your clothes in front of the mirror*”.

The magic shop window was the last scenario presented to the participants. This is the case that most impressed the participants. As in the previous cases, in the interactive shopping window, the combination of stickers and digital information guides people to use the functionalities in an immediately intuitive way.

Regarding utility, the group focused above all on the seller’s point of view: “*The magic mirror in the window is an effective way to capture customers and boost sales*”; “*The interactive window is very engaging and effectively invites people to enter*”; and “*Such a tool can make the difference for a shop*”.

The marketing expert added, “*There are people who would never enter some stores because they are out of their comfort zone. A showcase like this, however, would be an opportunity for them to try that brand without the embarrassment of entering the store, and, therefore, it is an opportunity to spread the brand name or convince new customers*”.

In the end, we asked the participants if there were any additional unexplored elements that could enhance their experience with a magic mirror. An observation shared by the group was the possibility to start the shopping experience at home by performing some setting activities, such as body measurement or selecting garments in an online catalog before going to the store to enjoy the AR experience.

In order to speed up shopping activities, a payment function integrated into the mirror or the possibility of finding the garments at the checkout looked useful to all.

To make the experience more interesting, a digital assistance system was proposed for giving advice on colors, the kind of fabric to wear given the weather, etc.

### 5.5. Results

The focus group results were examined using a thematic analysis method [43] suitable to analyze focus group activities about user experiences [44]. It consists of coding data and searching for themes and categories.

The categories resulting from our focus group are derived from the researchers’ interpretation of the collected data (research-denoted concepts). The approach used is *emergent coding*, which consists of analyzing the transcripts without a framework or a predetermined model to guide the analysis, giving the researchers the task of extracting a coherent model capable of capturing the important details.

Study validity was achieved by the researchers through a cross-checking activity of the focus group transcripts. Moreover, we adopted a reliability analysis based on Cohen’s Kappa [45]. It rates interrater reliability from 0 to 1, where 1 means perfect reliability between the coders. Two researchers were in charge of coding the focus group results following a set of coding instructions. A Kappa coefficient bigger than 0.6 was considered satisfactory, and bigger than 0.8 was considered a near-perfect agreement. The Kappa coefficient is calculated as follows:

K = (P_a_ − P_c_)/(1 − P_c_) (1)

P_a_ is the percentage of coders’ agreement, and P_c_ is the percentage of agreement by chance.

At the final iteration of our coders, P_a_ was 0.82, P_c_ was 0.37, and K was 0.71, which indicates a good agreement among the coders.

The coding activity brought out three main categories that can influence the shopping experience while using an AR fitting room: (1) *Utility* (66 items), (2) *Curiosity and interest* (43 items), and (3) *Comfortable environment* (26 items).

**Figure 3:**
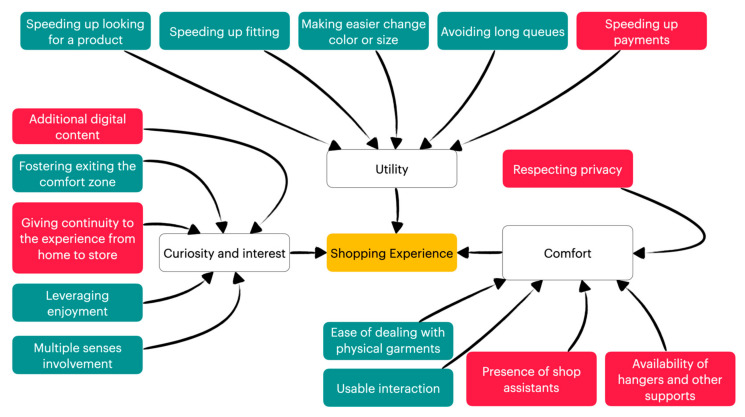
The three categories influencing the shopping experience and the elements that belong to it.

Figure 3 describes the three categories influencing the shopping experience and the factors that belong to it. The three categories are colored in white, and the factors belonging to them and those that are already supported by the patterns are in green. The factors in red are suggested by users but not yet supported.

Category 1 collects all the factors promoting the perception of utility, such as making the various shopping activities more efficient.

Category 2 collects the factors that stimulate the curiosity and interest of customers while using a magic window. More precisely, they are related to the involvement of various human senses, the enjoyment of being mirrored, being fostered to try brands or garments out of the comfort zone, but also to additional digital information leveraging the interest of the product and continuity of the experience from users’ home to the store.

The last category collects all the factors that can make the use of the mirror comfortable, such as the kind of interaction, the presence of shop assistants, hangers, and privacy when needed. Figure 3 shows that the patterns mainly support the Utility category, while they only partially support the other categories.

We can conclude that the interaction design patterns presented here are useful to support the development of engaging AR fitting rooms as a *meta-user interface* appropriate to interact with different shops environments. To enhance the shopping experience, designers should also not overlook aspects related to both the real different environments and the digital content that can increase the customers’ interest.

## 6. Conclusions

This work was oriented to study augmented reality technologies to implement *meta-user interfaces* to interact with fitting rooms or mirrors in a shop. In particular, starting from the analysis of the literature, of the systems present on the market and of the usability principles applicable to the context, it was possible to define and structure a set of interaction design patterns to develop AR fitting rooms that contribute to enhance customers’ shopping experience.

The patterns were then evaluated by a focus group with possible stakeholders to envision the potential shopping experience.

The results show that the produced patterns were positively evaluated, supporting a high level of perceived usability and utility, but also revealed a number of new factors that should be investigated and taken into consideration when designing an AR fitting room, possibly resulting in new patterns to the be added to the current list.

For the future, we are developing an AR fitting room prototype to be installed in a store and in various settings in order to validate the patterns in the field.

## Figures and Tables

**Table 1:** Desktop Components.

Component	Name
Central Processing Unit (CPU)	Quad-Core @ 3.4 GHz or better
Random Access Memory (RAM)	16 GB dual channel
Graphics Processing Unit (GPU)	Nvidia RTX 2070 or better
Storage	SSD 2.5″

**Table 2:** Structure of the pattern.

Explanatory Title	
Problem	It is the description of the problems related to the use of the system relevant to the usability and therefore to the experience of the stakeholders
To be used when	This section describes a situation where the interaction design pattern is appropriate to address the problem
Solution	It describes the design solution addressing the problem
Strength of evidence	A score from 1 (minimum) to 5 (maximum) indicating the strength of evidence of the patterns
Example	A picture showing an example of application of the pattern

**Table 3:** Body measurement pattern.

Body Measurement	
Problem	Making easy the user’s body measurement to start the AR fitting experience.
To be used when	When the user explicitly intents to use the magic mirror.
Solution	*The screen shows a silhouette in which the user must position himself. To make the task easier and more precise, a placeholder sticker can be installed on the floor.* *The silhouette must show a slight animation to let the user understand its interactivity.* *Once the user takes place in the silhouette, the system should play a confirmation sound as feedback and show the progress of the measurement operation via an animation. This is possible by means of a loading bar arranged on one of the sides of the template (horizontal bar below, vertical bar on the side), or by means of colors filling of the silhouette. Consider adding a verbal or textual message to inform the user.* *Text should appear at the head level to make it noticeable.* *Once the measurement is complete, a confirmation sound is played.* *Delete the graphics that are no longer needed.* *Be careful: the position of the pose for the measurement should be comfortable to the user. If the system requires a more tiring pose, this phase can be broken up into several steps.*
Strength of evidence	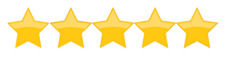
Example	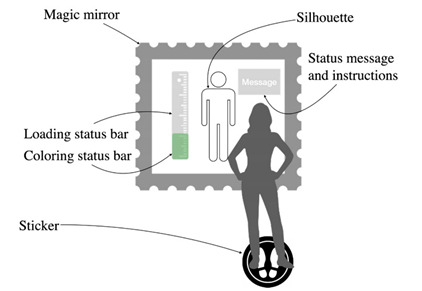

**Table 4:** Intuitive and comfortable interaction pattern.

Intuitive and Comfortable Interaction	
Problem	Making the interaction intuitive and comfortable.
To be used when	After the measuring task is complete.
Solution	*Because the system allows users to interact through a gestural interface guided by graphic elements present on the magic mirror, it is important to avoid constructing interactive gestures that can cause gorilla syndrome or that can be tiring for some users (such as the elderly). For these reasons, it is necessary to avoid interactions that require the arm to be fully extended or that require the arms to be held in a precise pose for a certain time.* *The final goal of a magic mirror is to allow users to fit a garment, not to interact with a complex interface. Then clearly separate the interaction flow into two stages:* *Interact with the catalog interface. The catalog interface could even overlap the user silhouette to make the user focus on the selection of the garment and relative characteristics.* *Garment fitting. Remove the main graphical interface to make the user focus only on the fitting task. Leave visible only controllers contextual to the garment.*
Strength of evidence	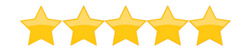

**Table 5:** Selecting the garment’s characteristics pattern.

Selecting the Garment’s Characteristics	
Problem	To allow users to select garment’s characteristics during the fitting in a comfortable way.
To be used when	While fitting a garment with different characteristics in colors or sizes.
Solution 1: *Garment* *interface menu*	*If the garment has optional characteristics, arrange small menu stripes close to the user’s elbow or shoulder.* *When a user moves the palm, a shadow follows the hand movement in the stripes* *The user makes a full fist on the garment to select it* *A sound and a slight animation are triggered as confirmation* *Since the fitting activity can take time and various users’ movements, it is advisable to allow them to adjust the menu height. This makes the menu interaction less tiring, as it can adapt the position over time. A graspable controller (by closing the fist) positioned just below the menu can be moved vertically, causing the menu slide effect.*
Solution 2: *Garment voice menu*	*When possible, allows the user to interact with the garment options using the voice besides the graphical interface. Example: “Size XL”, “Color Red”.* *When the user speaks, a hearing animation is shown in the mirror* *A sound and a slight animation are triggered as confirmation.*
Strength of evidence	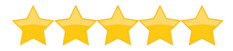
Example	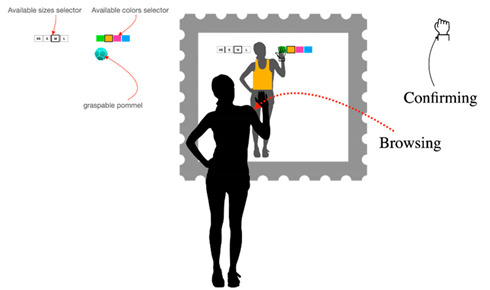

**Table 6:** Selecting the garment’s characteristics automatically.

Selecting the Garment’s Characteristics Automatically	
Problem	To allow users to select garment’s characteristics during the fitting and speeding up the task to avoid long queues.
To be used when	While fitting a garment with different characteristics in colors and when users need to speed up the fitting task. To use an alternative to Selecting the garment’s characteristics.
Solution	*The system automatically changes the characteristics of the product. It is important to display a message showing the name of the product every time it changes.*
Strength of evidence	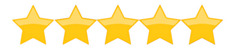
Example	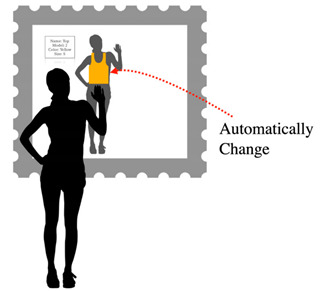

**Table 7:** Selecting a garment in the virtual catalog of the store.

Selecting A Garment in The Virtual Catalog of The Store	
Problem	To allow users to select through a virtual catalog the garment to fit.
To be used when	After the measuring task is complete.
Solution	*Consider making the catalog menu appear at user request. This allows leaving more screen space free to use as a mirror.* *Arrange the menu close to the user’s silhouette.* *Make the menu interaction easy and intuitive, for example using a stripe menu scrolling up and down like a wheel. This allows users to touch the stripe to the point they prefer. The stripe can be divided into two areas, one for scrolling and one for selecting the garment:* *a*. *The user shows the hand palm at the mirror center and makes a full fist to trigger the interface* *b*. *A shadow follows the hand movement* *c*. *When the palm touches the scroll area, the user can move the palm slightly upward or downward (alternatively, use the full fist). This causes the stripe to move upward or downward.* *d*. *When the palm touches a garment, this should stand out* *e*. *The user makes a full fist on the garment to select it* *f*. *A sound and a slight animation are triggered as confirmation* *g*. *The stage ends when the user selects the garment*
Strength of evidence	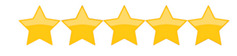
Example	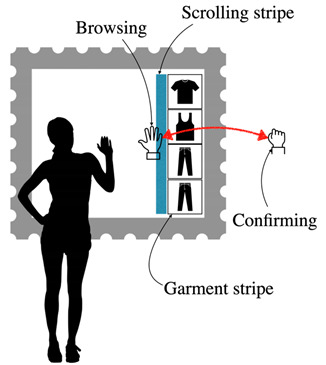

**Table 8:** Selecting a garment in the virtual catalog of the store using a second device.

Selecting a Garment in the Virtual Catalog of the Store Using a Second Device (Alternative to the Virtual Catalog)	
Problem	The system may allow users to choose the physical garment in the store
To be used when	After the measuring task is complete and in alternative to the virtual catalog.
Solution	*Since the interaction is divided into two tasks, fitting a garment and browsing a catalog, consider using a second device to physically divide the two tasks. This allows the user to focus easily and intuitively on each one. By deploying the catalog on a different device, you can provide both similar and different interactions.* *For example, you can use mid-air gestures or alternatively touch gestures, which are generally more familiar and intuitive for the user.*
Strength of evidence	* 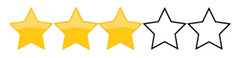 *
Example	* 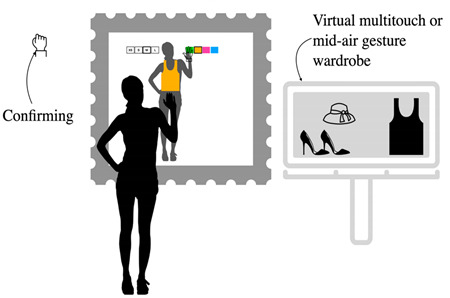 *

**Table 9:** Physically selecting a garment in the store.

Physically Selecting a Garment in the Store (Alternative to the Virtual Catalog)	
Problem	To allow users to choose the physical garment in the store
To be used when	After the measuring task is complete and an alternative to the virtual catalog
Solution	*A system increases its engagement when the user perceives it as simple, and useful for its purposes, when it provides a highly multimodal and natural interaction and is able to produce a pleasant experience.* *Taking these characteristics into consideration and considering that the user who enters a clothing store generally desires to physically touch the products, to discover them, to see them on mannequins, consider providing the user with a smart wardrobe next to the magic mirror. The wardrobe should be able to detect the product from an RFID or similar. Adding a product to the wardrobe is equivalent to selecting a product from the catalog to wear in AR.* *This allows you to maintain the positive aspects of the experience of visiting a shop, enriching it with the experience and functionalities in AR.* *You can apply any of the interaction design pattern related to the garment characteristics selection.*
Strength of evidence	* 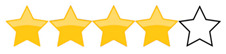 *
Example	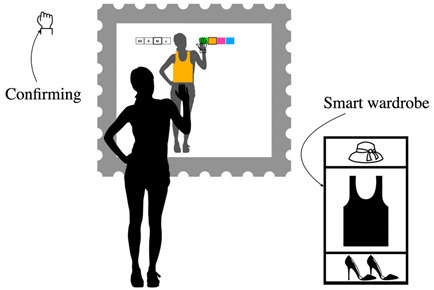

**Table 10:** Physically selecting a garment in the store using a mobile device.

Physically Selecting a Garment in the Store Using a Mobile Device	
Problem	To allow users to choose the physical garment in the store without carrying them through the store, for example, to respect health restrictions.
To be used when	After the measuring task is complete, instead of the virtual catalog and when health restrictions require customers to avoid touching the products in the store.
Solution	*The user experience must be continuous and consistent with the user’s real world. However, experiences from a specific domain different to the one under consideration can be successfully transmitted and adopted in the first domain.* *We refer to the one developed in many supermarkets that allows its customers to perform the so-called self-scanning using an app on their personal phone, save the products list, and then request to find them at the checkout. This makes a valuable and interesting mix of the online shopping experience, such as a web shopping cart, and the physical.* *Such an interaction paradigm can also be adopted for the AR fitting room. Customers can use the store app to select physical products in the store and fill their mobile shopping cart. Being easily identified, they can then approach the AR mirror, identify themselves with the app (using the simplest method for the context such as Bluetooth, RFID, etc …), and virtually try on the garments.* *You can apply any of the interaction design patterns related to the garment characteristics selection, integrating it with an alternative app interface. This allows novice or mobility impaired users to use the AR mirror more easily.* *The app also has an effect to be taken into consideration in the shopping business field: it can increase the customers’ loyalty and outline them.*
Strength of evidence	* 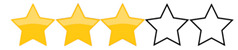 *
Example	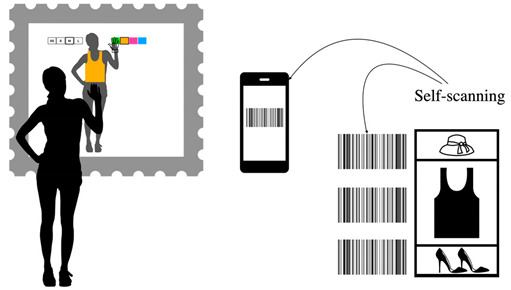

**Table 11:** Magic shop window patterns.

Magic Shop Window	
Problem	To draw attention of passers-by and to allow them to fit garments in the shop window
To be used when	When the mirror is positioned in the shop window
Solution	*Take advantage of the presence of the shop window to design a mid-air gesture interaction for the system but that which is perceived as a touch interaction by users.* *Place “interactive” stickers on the shop window to guide users to discover the gestures to perform with the mirror. The stickers must prompt users to place their palms in a specific place. This allows them to correctly guess the gestures, while the system recognizes them easily. Furthermore, when the user places the palm on the glass, the gesture is perceived as less tiring, and therefore it is possible to ask users to remain in a pose for a longer time.* *For example, place a sticker on the walk in front of the window, inviting people to stand there. Place two circle stickers on the glass at the average height of a person’s elbows. The stickers invite the user to touch them one by one with the palm. Touching the sticker, the system selects a specified garment in the shopping window. There is no need to explain to users the meaning of each sticker; they will learn it pleasantly with a trial-and-error paradigm.* *You can also place multiple stickers with the same meaning but at different heights to make them usable by people of different heights.*
Strength of evidence	* 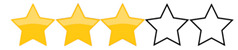 *
Example	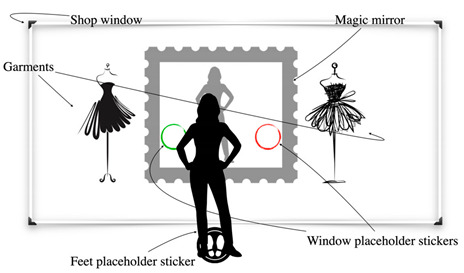

## Data Availability

The data that support the findings of this study are available from the corresponding author, [M.R.], upon reasonable request.
